# Lipoprotein Profile in Aged Rats Fed Chia Oil- or Hydroxytyrosol-Enriched Pork in High Cholesterol/High Saturated Fat Diets

**DOI:** 10.3390/nu10121830

**Published:** 2018-11-26

**Authors:** Jorge Arturo Santos-López, Alba Garcimartín, Sara Bastida, Mirandeli Bautista-Ávila, María José González-Muñoz, Juana Benedí, Francisco José Sánchez-Muniz

**Affiliations:** 1 Departamento de Farmacología, Farmacognosia y Botánica, Facultad de Farmacia, Universidad Complutense de Madrid, Plaza Ramón y Cajal s/n, 28040 Madrid, Spain; jorgears@ucm.es (J.A.S.-L.); a.garcimartin@ucm.es (A.G.); jbenedi@ucm.es (J.B.); 2 Departamento de Nutrición y Ciencia de los Alimentos, Facultad de Farmacia, Universidad Complutense de Madrid, Plaza Ramón y Cajal s/n, 28040 Madrid, Spain; sbastida@ucm.es; 3 Área Académica de Farmacia, Instituto de Ciencias de la Salud, Universidad Autónoma del Estado de Hidalgo, Ex Hacienda la Concepción s/n, Ctra. Pachuca-Tilcuautla, Hidalgo 42060, Mexico; mirandeli@hotmail.com; 4 Departamento de Ciencias Biomédicas, Unidad Docente de Toxicología, Facultad de Farmacia, Universidad de Alcalá, Ctra. Madrid-Barcelona km, 33,600, 28805 Alcalá de Henares, Spain; mariajose.gonzalez@uah.es

**Keywords:** aging, Chia oil, hydroxytyrosol, restructured pork, lipoprotein

## Abstract

Restructuring pork (RP) by adding new functional ingredients, like Chia oil (one of the richest natural source of α-linolenic acid) or hydroxytyrosol (HxT) (potent antioxidant), both with hypolipidemic activities, is one of the strategies that may help to reduce the potential negative effects of high meat products consumption. The aim of this study was to evaluate the Chia oil- or HxT-enriched-RP effect on the lipoprotein profile of aged rats fed high-fat, high-energy, and cholesterol-enriched diets. RP samples were prepared by mixing lean pork and lard with or without Chia oil (152.2 g/kg fresh matter) or HxT (3.6 g/kg fresh matter). Diets were prepared by mixing a semisynthetic diet with freeze-dried RP. Groups of 1-year male Wistar rats were fed the following experimental diets for 8 weeks: C, control-RP diet; HC, cholesterol-enriched-RP diet; and Chia oil-RP (CHIA) and HxT, Chia oil- or hydroxytyrosol-RP, cholesterol-enriched diet. Plasma lipid, lipoprotein profile, SREBP-1c protein, and low-density lipoproteins (LDL) receptor gene (*Ldlr*) expressions were evaluated. Compared to C diet, the HC diet increased plasma cholesterol, triglycerides, free fatty acids, total lipids, and SREBP-1c expression, but reduced *Ldlr* expression and significantly modified the lipoprotein profile, giving rise to the presence of high levels of atherogenic cholesterol-enriched very low-density lipoproteins (VLDL) particles. Compared to the HC diet, the HxT diet did not produce significant changes in feed intake but it reduced the body weight. Chia oil and HxT partially arrested the negative effects of the high-fat, high-energy, and cholesterol-enriched meat-based diets on lipemia and lipoproteinemia, mostly by reducing the amount of cholesterol content in VLDL (60% and 74% less in CHIA and HxT vs. HC, respectively) and the VLDL total mass (59% and 63% less in CHIA and HxT vs. HC, respectively). Free fatty acids (FFA) significantly correlated with adipose tissue weight and VLDL total mass (both *p* < 0.05), and plasma triglycerides, phospholipids, total lipids, and SREBP-1c (all *p* < 0.001), suggesting the important role of FFA in lipoprotein metabolism. Results support the recommendation to include these ingredients in pork products addressed to reduce the presence of increased atherogenic particles in aged people at CVD risk consuming large amounts of pork.

## 1. Introduction

Pork and pork products are highly consumed foods in developed countries [1]. Even though they are a great source of protein, vitamins, and minerals, there is significant epidemiological evidence that correlates meat consumption with degenerative disease, like cardiovascular disease (CVD) [1] or an imbalance in lipid profile. Therefore, there has been growing interest in the research field that evaluates quantitative and qualitative modifications to obtain functional meats and meat products [2,3]. Restructuring pork (RP), by partially adding or changing some of the meat components, would permit to incorporate active ingredients with potential functional effects [2,3]. Our research group has previously studied the effect of walnut-enriched meat products consumption on the antioxidant and lipoprotein profile of volunteers at CVD risk [2]. In addition, the consumption of omega-3-enriched-reduced fat meat products partially reduced thrombogenesis, coagulation, and insulin-resistance markers [4]. Moreover, we have evaluated the effect of seaweed-enriched-RP consumption on different CVD risk markers in cholesterol-fed rats. Results largely depended on the type and composition of seaweed included [5].

It has been recognized that adjustments in the quality of dietary lipids, such as omega-3 polyunsaturated fatty acids (ω-3 PUFA), are important in the prevention of metabolic disorders [6]. In this regard, interest in α-linolenic acid (ALA; 18:3 ω-3) as a functional ingredient has grown in recent years because of its association with improvements in plasma lipid concentrations and CVD [7]. Nevertheless, there are few foods that contain adequate amounts to provide the benefits associated with ALA consumption [8].

The seeds of *Salvia hispanica* L., known as chia seeds, are one of the richest sources of ALA found in nature [7,8]. The lipid content varies from 60–80% of total lipids comprised of ALA and 40–20% of linoleic acid (ω-6). Rodent studies have proved that the intake of Chia oil may lower serum cholesterol, low-density lipoproteins (LDL), and triglycerides, and increase high-density lipoproteins (HDL) [9]. Furthermore, other studies have suggested an improvement in adiposity, blood lipids, and insulin resistance in dyslipidemic rats after *Salvia hispanica* treatment [10]. The mechanism of this plasma lipid improvement for dietary chia is not completely understood. It is possible that ALA, as an eicosapentaenoic (EPA) and docosahexaenoic (DHA) acids precursor, could play an important role in fatty acid metabolism in the liver, such as lipogenesis and fatty acid oxidation [11].

Hydroxytyrosol (HxT) is a polyphenol that has been used as a functional ingredient, especially in PUFA-enriched meats, because of its powerful antioxidant capacity [12], and in pre-cooked meat products enriched with ω-3 PUFA [13]. Its antioxidant properties in experimental animals, when incorporated into RP in high-fat diets, have already been evaluated by our group [14]. Additionally, it has been found that a reduction of triglycerides and LDL-cholesterol, and an increase of HDL-cholesterol levels, have been reported in diabetic rats [15]. Cao et al. demonstrated the protective effect of supplementing diet with HxT in a high-fat-diet animal model that induced obesity, hyperglycemia, hyperlipidemia, and insulin resistance. This study proved the ability of HxT to decrease lipid accumulation [16]. Although much information about the impact of omega-3 PUFA on lipids is available, their effects on lipoprotein number and composition have been scarcely reported [17]. High-energy, saturated fat, and cholesterol diets have been proven to induce nonalcoholic steatohepatitis in aged rats [14,18]. A previous study of our group with Chia oil and HxT supplementation has demonstrated the antioxidant and anti-inflammatory effects of these ingredients [19]. The results showed the protective effect of the experimental diets that reduced the oxidative damage through the regulation of glutathione levels by increasing glutathione reductase and decreasing glutathione peroxidase levels. Also, Chia oil supplementation, despite its high PUFA content, was able to reduce lipid peroxidation. Additionally, the findings suggested that Chia oil and HxT were exogenous stimuli that promoted the activation of the Nrf2 pathway to control the pro-oxidative response to dietary cholesterol and ageing [19]. However, to the best of our knowledge few papers have tested how the inclusion of HxT or Chia to this animal model of nonalcoholic steatohepatitis affects the lipoprotein content and composition.

This study hypothesizes that Chia oil or HxT incorporated into a RP partially block the changes on lipoprotein profile induced by a high cholesterol/high saturated fat diet. In addition, the aim of this investigation was to determine the effects of the different experimental diets on lipoprotein profile and composition, SREBP-1c protein, and LDL receptor (*Ldlr*) gene expressions in aged rats.

## 2. Material and Methods

### 2.1. Animals and Diets

All experiments were performed in compliance with Directive 86/609/EEC of 24 November 1986 (amended by Directive 2003/65/EEC of 22 July 2003) on the protection of scientific research animals and approved by the Spanish Science and Technology Advisory Committee (project AGL 2011-29644-C02-02) and by the Ethics Committee of the Universidad Complutense de Madrid (Spain). The experimental diets were prepared by modifying its composition to ensure high saturated fat/high cholesterol/high energy content (Table 1). Three of these diets were supplemented with hypercholesterolaemic inductors (cholesterol plus cholic acid). Chia oil and hydroxytyrosol (HxT, 2-(3,4-dihydroxyphenyl)-ethanol, SeproxBiotech, Madrid, Spain) were used in diet preparation. Previous studies have evaluated the HxT effects at different concentrations as food ingredients. According to these investigations, we decided to use this component in a higher amount than the regular intake provided by diet (i.e., olive oil) without reaching toxic concentrations [14,20,21]; for Chia oil we used a concentration that does not exceed dietary recommendations but that completely substitutes pork lard in the RP. Lean pork and lard were acquired at local production stores. Lean pork (849 g) and fat (151 g) were homogenized, and restructured pork (RP) was prepared following previously described protocols [22]. For Chia-RP, Chia oil (152.2 g/kg fresh matter) was homogenized with lean pork and the rest of the components. In the case of HxT-RP, hydroxytyrosol (3.6 g/kg fresh matter) was first homogenized with lard and then mixed with the rest of components. The different RP mixtures were freeze-dried in a LyoAlfa 10 freeze dryer (Telstar, Terrassa, Spain). The four semi-synthetic experimental diets were prepared as (1) high saturated fat control-RP (C) diet without added cholesterol; (2) high saturated fat and high cholesterol control diet (HC) (here the maize starch was substituted by 1.26% cholesterol and 0.25% cholic acid); (3) and (4) chia- and HxT-RP high saturated fat and high cholesterol diets (CHIA and HxT, respectively), similar to HC but incorporating Chia oil and HxT. To prepare the final experimental diets, 217 g of RP and 783 g of the modified semi-synthetic formulation (Panlab S.L., Barcelona, Spain; reference U8959 version 180) were mixed by serial dilutions until homogeneity (Table 1). Diets were calculated to accomplish micronutrient requirements at their final concentration (Supplementary Table S1).

### 2.2. Experimental Design

The experimental animals were obtained from Harlan Laboratories Models, SL. (Barcelona, Spain). The twenty four male Wistar rats were housed individually under controlled conditions (22.3 ± 1.8 °C and 12 h light-dark cycle). Rats were fed one week with commercial pellets (Panlab, Barcelona, Spain) as adaptation period. Water was provided ad libitum. When the animals reached one year old and 500 g weight they were distributed into four groups of six animals each and fed the experimental diets for eight weeks. It is well known throughout several previous studies that three to five weeks of cholesterol feeding are long enough to assess effects on lipoprotein composition by increasing atherogenic particles. Previous research has demonstrated that omega-3 fatty acids reduce VLDL synthesis [24,25], and there is evidence of the positive HxT effect on CVD biomarkers [26] in similar feeding periods. A daily register of food consumption and weekly body weight measurement was performed. No unexpected deaths were registered along the study. Rats were euthanized one at the time from each of the four groups by extracting blood by heart puncture, previously anesthetized with isoflurane (5%). Then, liver and visceral adipose tissue (mesenteric, epididymal and perirenal) were collected and nitrogen was frozen and kept at −80 °C until analysis.

### 2.3. Lipoprotein Isolation

Plasma was separated from cell portion by centrifugation for 20 min at 2500× rpm (1500× *g*) and kept at 4 °C until analysis. The lipoprotein fractions were obtained following the Terpstra et al. method [27] with slight modifications [28] from 2 mL plasma by saline gradient ultracentrifugation for 22 h at 40,000× rpm (Beckman L8-70M, Palo Alto, CA, USA) with the SW-40.1 rotor. Lipoproteins were isolated according to the conventional limitations for rats based on the density range (VLDL (ρ_20_ < 1.0063 g/mL), intermediate-density lipoproteins (IDL) + LDL (1.0063 < ρ_20_ < 1.057 g/mL) and HDL (1.057 < ρ_20_ <1.21 g/mL)) [29].

### 2.4. Plasma Lipid Analysis and Lipoprotein Composition

The total cholesterol, phospholipids, and triglycerides levels were quantified in plasma and lipoprotein fractions (VLDL, IDL + LDL, and HDL) using standard enzymatic colorimetric tests (Spinreact S.A., Girona, Spain) in a T80 ϕ spectrophotometer from PG Instruments (PG Instruments Ltd. Wibtoft, Leics, UK). Given that the cholesterol determination using Spinreact protocol includes the cholesterol esterase/cholesterol oxidase/peroxidase enzyme mix, the separately determination of free and esterified cholesterol was not assessed. Thus, total cholesterol was the result of both the preexisting free cholesterol plus the free one formed after the enzymatic reaction with esterified cholesterol. Total lipids were calculated as the sum of total cholesterol, triglycerides, and phospholipids [28], and atherogenic index was calculated as (total cholesterol − HDL-cholesterol)/HDL-cholesterol. Protein content of isolated lipoproteins was determined using the Bradford [30] method. The total mass of each lipoprotein fraction was calculated as the sum of lipids (total cholesterol + triglycerides + phospholipids) and proteins, all in mg/dL [28].

### 2.5. Extraction and Analysis of RNA and Quantification by Reverse Transcription-Polymerase Chain Reaction (RT-PCR)

Total RNA was isolated from liver (100 mg) with Trizol (Sigma, Barcelona, Spain) according to the manufacturer’s instructions. 1 µg RNA was reverse-transcribed to first-strand cDNA using a revert aid H minus first strand cDNA synthesis kit (Thermo Fisher Scientific, Waltham, MA, USA). Relative *Ldlr* mRNA levels were quantified using a LightCycler™ Real Time PCR Detection System (Roche diagnostics, Indianapolis, IN, USA), and SYBR-Green (Biotools, Madrid, Spain) as binding dye for fluorescence detection.

The following PCR conditions were used: pre-incubation at 95 °C for 10 min followed by 45 cycles of denaturation at 95 °C for 10 s, with an annealing temperature of 67 °C for 10 s, extension 72 °C for 15 s, and cooling 40 °C for 30 s. Primers sequences: CTGTATTCACGGTAGCCGCC (forward) and TGGGTCACATTGATGCAGCC (reverse). All sample mRNA levels were normalized to β-actin values and the results expressed as fold changes of threshold cycle (Ct) value relative to C rats using the 2^−∆∆Ct^ method [31].

### 2.6. Protein Expression by Western-Blot Analysis

Liver protein lysate was obtained and separated in 10% sodium dodecyl sulfate-poly-acrylamide gel electrophoresis (SDS-PAGE). Gel was then blotted onto PVDF Amersham Hybond-P membrane (GE Health-care, Buckinghamshire, UK) and incubated with its corresponding antibodies (anti-SREBP-1c (sc-366), and anti-TBP (sc-273) from Santa Cruz Biotechnology, Dallas, TX, USA). TATA-binding protein (TBP) was used as loading control. Blot was developed by enhanced chemiluminescence using an Amersham ECL Plus Western Blotting Detection Reagent (GE Health-care, Buckinghamshire, UK) according to manufacturer’s instructions.

### 2.7. Statistical Analyses

Statistical analyses were performed using the SPSS version 19.0 statistical analysis package (SPSS, Inc., Chicago, IL, USA). Results were expressed as means and standard deviations. One way analysis of variance (ANOVA) followed by T2 of Tamhane post hoc test was applied. Pearson product-moment correlations were performed to assess linear dependence between variables. Differences were accepted as significant at *p* < 0.05 (a > b > c).

## 3. Results

### 3.1. Feed Consumption, Body Weight, Adipose Tissue Weight, Fecal Excretion, and Diet Digestibility

Initial body weight and feed intake were not significantly modified between the different groups (*p* = 0.094 and *p* = 0.058, respectively). The rest of the parameters shown in Table 2 were significantly affected by the different experimental diets (all *p* < 0.001). Final body weight and the percent of body weight gain were significantly lower in HC vs. C rats (*p* < 0.001). HxT group induced lower final body weight and body weight gain than CHIA and HC animals (*p* < 0.001). Significant differences were shown in the cholesterol intake, fecal excretion, fecal fat, and dietary digestibility (all *p* < 0.001). The cholesterol intake was higher in HC, CHIA, and HxT rats than in C animals (all *p* < 0.001). Significant increase in fecal excretion, fat, and cholesterol, and lower dietary digestibility were observed (all *p* < 0.001) in HC vs. C groups. The HxT diet significantly decreased dietary digestibility (*p* < 0.001) with respect to the C diet. CHIA diet when compared to the C diet displayed higher fecal fat content (in dry matter) (*p* < 0.001). All cholesterol-enriched diet groups significantly increased fecal cholesterol (*p* < 0.001). C animals showed the highest adipose tissue weight and adiposomatic index (*p* < 0.001) (Table 2).

### 3.2. Plasma and Liver Lipids and Atherogenic Index

Table 3 shows the plasma lipid composition, the atherogenic index, and the cholesterol/phospholipid ratio in the experimental groups. All parameters were significantly affected by the different diets (*p* < 0.001). HC rats displayed significantly higher plasma cholesterol, triglycerides, total lipids (*p* < 0.001), free fatty acids, and atherogenic index than C animals. CHIA rats showed lower values for all plasma lipids (*p* < 0.001) than HC animals, and even lower triglyceride, phospholipid, and total lipid levels than C (*p* < 0.001). The CHIA diet also reversed the effect of HC on total cholesterol and total lipids, reaching same values, and even lower for free fatty acids, as C diet. Plasma triglycerides, phospholipids, and total lipids were significantly lower (*p* < 0.001) in HxT compared to HC rats, while the atherogenic index was higher (*p* < 0.001) when compared to C animals. Non-significant differences except for phospholipids were found between CHIA and HxT groups. The total liver cholesterol was significantly higher in HC than C rats. HxT significantly lowered liver cholesterol in comparison to HC and CHIA (at least *p* < 0.01).

### 3.3. Plasma Lipoprotein Composition and Profile

The different lipoprotein fraction compositions are shown in Table 4. Most components of VLDL, IDL + LDL, and HDL fractions were significantly affected by diets (ANOVA *p* < 0.001), except HDL-triglycerides (ANOVA *p* = 0.059). HDL was the highest cholesterol transporters in C rats (approximately 80%), while VLDL were the major carrier of triglycerides (approximately 50%). Dietary inclusion of the experimental diets significantly altered all the studied lipoprotein parameters (ANOVA, *p* = 0.01). All components of VLDL and IDL + LDL fractions were higher in HC than in C rats (*p* < 0.001). By the contrary, all components of HDL fraction with the exception of triglycerides (*p* > 0.05) were significantly lower in HC than C animals. CHIA and HxT diets normalized all VLDL compounds, reaching the same levels as C rats, except protein that was significantly higher than C but lower than HC (*p* < 0.001). Triglycerides in the IDL + LDL fraction were significantly lower in CHIA and HxT than in HC rats (*p* < 0.001). The CHIA and HxT diets did not significantly affect (*p* > 0.1) any HDL compounds in comparison to HC diet.

**Figure 1:**
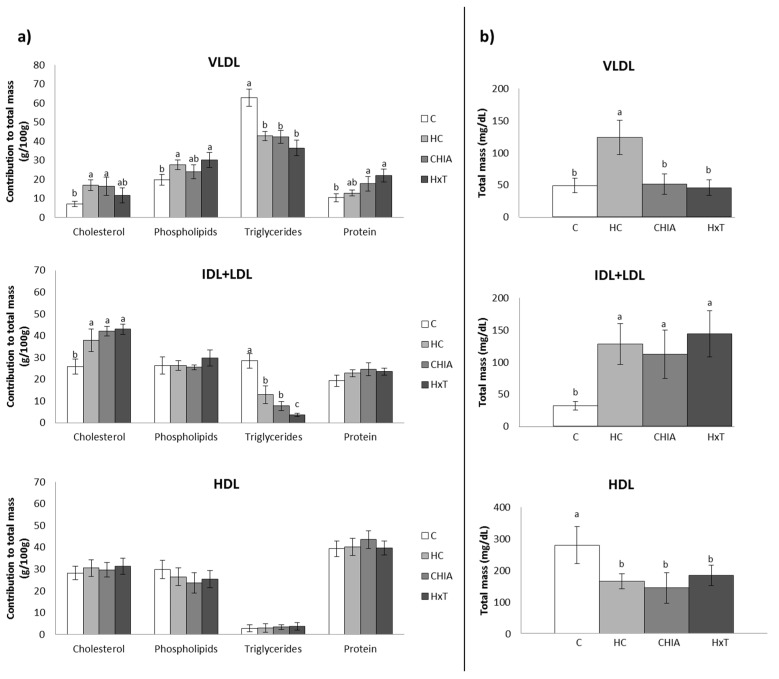
(**a**) Percentage of contribution of the different lipids and protein to the total mass of plasma VLDL, IDL + LDL and HDL. (**b**) Total mass (total lipids + protein) of the different lipoproteins. C, control-RP diet; HC, Cholesterol-enriched high-saturated/high-cholesterol control-RP diet; HxT, hydroxytyrosol-RP cholesterol-enriched high-saturated/high-cholesterol diet; CHIA, Chia oil-RP cholesterol-enriched high-saturated/high-cholesterol diet. VLDL, very low-density lipoproteins; IDL, intermediate-density lipoproteins; LDL, low-density lipoproteins; HDL, high-density lipoproteins. Bars, mean values ± SD, for a variable bearing different letters indicate significant differences between groups for each parameter (*p* < 0.05; a > b > c) (one way ANOVA followed by the T2 Tamhane post-hoc test).

The contribution of lipids and proteins to the total mass of plasma VLDL, IDL + LDL, and HDL fractions is shown in Figure 1a,b. All VLDL components and IDL + LDL-cholesterol and triglycerides were significantly affected by diet (ANOVA *p* < 0.05). VLDL in HC rats contained significantly higher total cholesterol and phospholipids but lower triglyceride amount (*p* < 0.001) with respect to their C counterparts. HxT diets tended to reduce the cholesterol contribution to the VLDL total mass relative to HC (*p* = 0.028). CHIA and HxT rats displayed significantly higher contribution of proteins to total VLDL mass than their C equivalents (*p* < 0.001). Total cholesterol contributed higher (*p* < 0.001) and triglycerides lower (*p* < 0.001) to the total mass of IDL + LDL in HC, CHIA, and HxT vs. C rats. Triglycerides contributed less to the IDL + LDL mass in HxT than in CHIA or HC (*p* < 0.05). There were no differences in lipids and protein contributions to the HDL mass between groups (all *p* > 0.1) (Figure 1a).

All total mass values in the different lipoproteins were significantly affected by experimental diets (*p* < 0.001). In comparison to C rats, HC showed higher total mass in VLDL and IDL + LDL but lower in HDL fraction. When comparing CHIA and HXT with HC, a significant decrease (*p* < 0.001) in total VLDL mass compared to values similar to the C animals was observed (Figure 1b).

**Figure 2:**
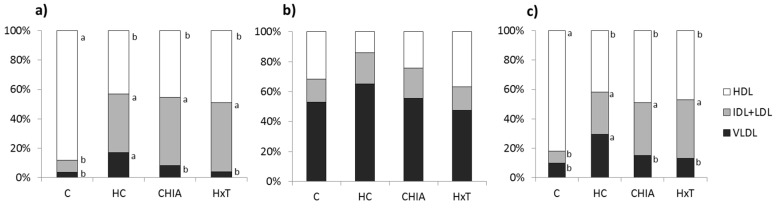
Lipid percentage transport in the different lipoproteins: VLDL, IDL + LDL, and HDL, in rats fed the experimental diets. (**a**) Cholesterol, (**b**) triglycerides, and (**c**) phospholipids. C, control-RP diet; HC, cholesterol-enriched, high-saturated/high-cholesterol, control-RP diet; HxT, hydroxytyrosol-RP, cholesterol-enriched, high-saturated/high-cholesterol diet; CHIA, Chia oil-RP, cholesterol-enriched, high-saturated/high-cholesterol diet. VLDL, very low-density lipoproteins; IDL, intermediate-density lipoproteins; LDL, low-density lipoproteins; HDL, high-density lipoproteins. Mean values within the same lipoprotein for C, HC, CHIA, or HxT with unlike letters were significantly different (*p* < 0.05, T2 Tamhane test), represented by vertical areas (a > b > c).

Figure 2 displays the information about the specific lipid percentage transport in the different lipoproteins. Cholesterol and phospholipid relative transport via lipoproteins was affected by the experimental diets (at least *p* < 0.01). Total cholesterol and phospholipids contribution in HC rats for VLDL and IDL + LDL was significantly higher but lower for HDL (*p* < 0.001) than the C rats. CHIA and HxT displayed a lower (*p* < 0.05) VLDL contribution to the total cholesterol and phospholipids transport when compared to HC animals.

### 3.4. Liver Ldlr Gene and SREBP-1c Protein Expressions

*Ldlr* (ANOVA *p* < 0.001) and SREBP-1c (ANOVA *p* < 0.05) were significantly affected by diets. HC rats presented significantly lower (*p* < 0.05) *Ldlr* expression than C rats (Table 5). *Ldlr* expression did not show significant differences between HC, CHIA, or HxT. The SREBP-1c protein expression was significantly higher in HC than in the C group. CHIA and HxT significantly reduced the expression to even lower values than HC and C (at least *p* < 0.05).

### 3.5. Pearson Product-Moment Correlation Between Variables

Table 6 shows only relevant correlations (*p* < 0.01) between the analyzed variables. Adipose tissue weight positively correlated with plasma triglycerides and phospholipids, *Ldlr*, SREBP-1c, and HDL total mass, and negatively correlated with cholesterol intake and excretion, atherogenic index, and IDL + LDL total mass. FFA positively correlated with plasma triglycerides, phospholipids and total lipids, SREBP-1c expression, and VLDL total mass. *Ldlr* negatively correlated with intake, fecal, and total liver cholesterol. SREBP-1c positively correlated with plasma triglycerides, phospholipids, total lipids, and VLDL total mass. The atherogenic index positively correlated with IDL + LDL total mass and negatively with HDL total mass.

## 4. Discussion

Quantitative and qualitative modifications of meat composition are emerging topics addressed to decrease the potential negative effects of a high meat products consumption on CVD [2,3]. The major aim of this study was to characterize the potential benefits of consuming Chia oil- or HxT-enriched pork on lipemia, lipoproteinemia, and lipoprotein profile of aged rats fed high-fat, high-energy, cholesterol-enriched diet. The latter diet has been reported to induce nonalcoholic steatohepatitis in aged rats [14,18]. Chia oil or HxT partially reversed alterations induced by cholesterol feeding, through normalizing VLDL total lipids and reducing VLDL proteins, as well as by modifying SREBP-1c and *Ldlr* expressions, which are two important markers of lipid metabolism.

**Figure 3:**
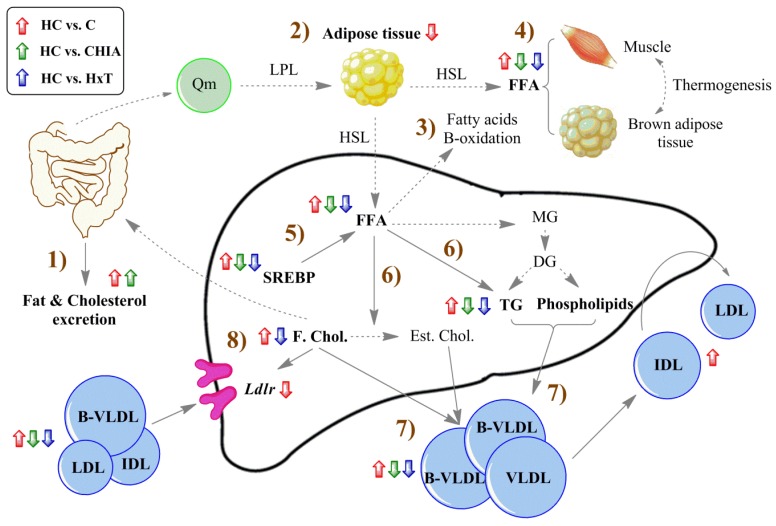
Proposed mechanism of the effect of the experimental diets on lipid and lipoprotein metabolism. Different colored arrows (red, green, or blue) indicate the increase (↑) or decrease (↓) for each marker assigned. Points: (**1**) cholesterol feeding increased fat and cholesterol excretion; (**2**) the decrease of dietary digestibility induces a reduction in body and adipose weights, with HSL activation and FFA release from adipose tissue; (**3**) Chia oil decreased liver FFA availability through the β-oxidation activation; (**4**) HxT decreased liver FFA availability through the activation of the thermogenesis process; (**5**) CHIA and HxT decrease SREBP-1c protein expression, contributing to a lower FFA amount; (**6**) FFA increase by cholesterol feeding, induced higher liver amount of TG, phospholipids, and esterified cholesterol; (**7**) the TG, phospholipids, and esterified cholesterol increase gave rise to the B-VLDL production. The lower FFA amount in CHIA and HxT induced lower lipid production and thus lower B-VLDL; (**8**) the liver cholesterol increase causes a reduction in *Ldlr* expression. CHIA and HxT, by reducing liver lipid storage, tended to increase *Ldlr* expression that in turn contributed to a reduction of atherogenic particles (B-VLDL, LDL, and IDL). C, control-RP diet; HC, cholesterol-enriched, high-saturated/high-cholesterol, control-RP diet; HxT, hydroxytyrosol-RP, cholesterol-enriched, high-saturated/high-cholesterol diet; CHIA, Chia oil-RP, cholesterol-enriched, high-saturated/high-cholesterol diet. VLDL, very low-density lipoproteins; IDL, intermediate-density lipoproteins; B-VLDL, cholesterol- enriched, very low-density lipoprotein; LDL, low-density lipoproteins; HDL, high-density lipoproteins; Qm, chylomicrons. LPL, lipoprotein lipase; HSL, hormone-sensitive lipase; FFA, free fatty acids; MG, monoglycerides; DG, diglycerides; TG, triglycerides; F. Chol., free cholesterol; Est. Chol., esterified cholesterol. SREBP, sterol regulatory element-binding protein-1c; *Ldlr*, low density lipoprotein receptor.

Based on the evidence of the present and previous works, we propose a probable mechanism that explains, on one hand, the effects of adding cholesterol to a high fat/high energy RP-diet and, on the other hand, the effects of including Chia oil and HxT in those RP-diets (Figure 3).

The C rats demonstrated a lower dietary intake than that of growing rats that received seaweed-RP diets [5]. However, results were similar to those observed by Nesic et al. [32], which can be partially related to the ageing anorexic effect [33]. There were no significant differences between feed intake and body weight in the HC group, in comparison with results for younger cholesterol-fed rats [5,22,29]. Thus, although body weight gain was significantly reduced in all rat groups fed the experimental cholesterol-enriched diets, aging may alter the effect of cholesterol intake on body weight. Nevertheless, the findings could also be a result of the high-energy/high-saturated fat content of experimental diets. As in cholesterol-fed animals [34], HC rats registered higher fecal excretion than C rats (Figure 3, point 1), suggesting a reduction in diet digestibility of the HC animals that would contribute to both a lower body weight gain and adipose tissue mass. A significant negative correlation was found between body weight gain and fecal fat deposition (*p* < 0.01), which supports previous explanations and could explain the decrease of final body weight in the cholesterol fed animals (Figure 3, point 2). Our group has reported that the dietary cholesterol supplementation in rats decreased the adipose tissue stores by increasing the hormone sensitive lipase (HSL) expression, a mechanism linked to plasma cholesterol regulation [35].

In agreement with the aging effects observed in non-cholesterol fed rats [36], C rats registered moderately higher plasma cholesterol and phospholipids levels than younger rats [5,28,34]. In fact, 37.5% of C animals presented cholesterolemia values higher than 100 mg/dL, cut-off point of rat hypercholesterolemia [37]. Dietary cholesterol supplementation increased total lipids and cholesterol levels (19% and 32%, respectively). In addition, contingence test showed significant differences (*p* < 0.05) of distribution between hyper- and normocholesterolemic rats in HC and C groups, since all the HC rats were moderately hypercholesterolemic (>2.52 mmol/L).

Nonetheless, in comparison with previous studies carried out by our group in younger animals [5,24], the 0.8 mmol/L cholesterol increase due to dietary cholesterol + cholic acid was smaller than expected. Erdinçler et al. [36] also observed a small but substantial increase in plasma cholesterol in aged rats fed similar quantities of cholesterol. At a certain level, CHIA blocked the hyperlipidemic effect of cholesterol shown by HC group by means of lowering cholesterolemia, triglyceridemia, phospholipemia, total lipids, and FFA, by increasing fatty acid β-oxidation (Figure 3, point 3) [25]. These results are consistent with those of previous studies where chia administration, as a supplement of fiber and fat, reduced lipid parameters in rats [38].

With respect to HxT, the hypotriglyceridemic and hypophospholipemic mechanism could be related to the thermogenic properties of HxT, as proposed in previous studies [19,39]. The induced thermogenesis would decrease the lipid accumulation and increase the fat mobilization from adipose tissue, giving rise to a lower plasma FFA amount in these rats (Figure 3, point 4).

The antioxidant defense system improvement by CHIA and HxT, previously reported by our group, could be partially related to the hypolipemic effects found in the present paper, as it plays an important role in the reduction of lipid accumulation in the liver [19]. This information is relevant, as it is well known that fatty liver disease can be highly prevented through the antioxidant defense system activation [19].

C rats displayed a characteristic rat lipoprotein profile with low LDL and high HDL levels [5,27,34,37]. Rat has been categorized as HDL-animal [26,37], exhibiting a very effective uptake of VLDL through the *Ldlr* and a lesser apolipoprotein (apo) B transfer from VLDL to LDL. Nonetheless, it has been reported that age induces a lower *Ldlr* expression [40], which would in part explain the contribution of the IDL + LDL fraction to total plasma lipids in C rats.

HC rats showed increased VLDL fraction levels, likely due to their low Ldlr gene expression and high SREBP-1c protein expression. It has been reported that an elevation on SREBP-1c, expression occurs as a consequence of a reduction in the pool of hepatic free cholesterol, which in turn would depend on the availability of FFA in the liver [41]. Viejo et al. [42] found a marked increase in both hepatic cholesterol esters and the esterified/free cholesterol ratio in rats fed high-cholesterol diets. These authors suggested that this esterification would occur as a mechanism to maintain reduced levels of free cholesterol, thus attempting to maintain reduced levels of *Ldlr*. These considerations complied with the observed increase of SREBP-1c and FFA in the HC group (Figure 3, point 5).

A relevant mechanism linked to the hyperlipidemic effects of HC diet is explained through the high plasma FFA levels in those animals. FFA are one of the most powerful signals to induce liver triglyceride and other lipid synthesis, which are also linked to the decrease in adipose tissue weight [35] (Figure 3, point 6). Significant correlations between plasma lipids and VLDL total mass with FFA were found in the present paper.

Lipoprotein levels and composition suggest that there was a reasonably higher number of cholesterol-enriched VLDL in HC rats than in their C equivalents (Figure 3, point 7). The IDL + LDL fraction was also richer in cholesterol and poorer in triglycerides. In addition, the protein content of this fraction was higher, proposing that there was an elevated number of these particles in plasma. Vázquez-Velasco et al. [43] found that IDL + LDL increased in cholesterol-fed fa/fa rats. The results of our study agree with previous publications that reported the presence of cholesterol enriched-VLDL (β-VLDL) [28,44]. These β-VLDL have been defined as atherogenic lipoproteins for the rat [45]. The reduction of HDL-total mass in HC rats was similar to previous results in hypercholesterolemic rats [5,43], probably linked to HDL uptake by the scavenger receptor B-1 (SRB1) to increase cholesterol excretion via bile [46]. Since the HDL composition was unaffected but their total mass concentration was diminished, it can be accepted that there was a reduction in the number of HDL particles.

As in HC rats, the VLDL fraction in CHIA or HxT rats was cholesterol-enriched. However, both groups showed considerably minor levels of protein and total mass in the VLDL fraction, suggesting a clear reduction in the number of VLDL particles. This mechanism has not been completely understood, but it seems to involve a fall in the triglycerides and protein amounts available for the VLDL synthesis, indicative of the hypotriglyceridemic mechanism proposed for ω-3 fatty acids [25]. The high concentration of ω-3 fatty acids in Chia oil has been found to decrease VLDL levels [47]. It can be hypothesized that CHIA would reduce phospholipids, triglycerides, and therefore VLDL particles through ω-3 fatty acid β-oxidation [25] and by increasing the pool of fatty acids for liver cholesterol esterification [44], while HxT would increase the thermogenic process. CHIA or HxT did not increase *Ldlr* expression vs. HC rats, although it tends to increase by 33% and 22% for CHIA and HxT, respectively. This may be as a result of the capacity of CHIA or HxT to induce relocation of lipids in the body exerting a hepatoprotective effect. This is achieved by decreasing lipid accumulation in the liver and in visceral adipose tissue. In addition, VLDL synthesis is reduced making the increased expression of the SREBP-1c—a key marker in the lipogenesis pathway—unnecessary [48] (Figure 3, point 8).

## 5. Conclusions

In brief, this study has the merit of being the first to describe the effects that Chia oil- or HxT-enriched pork have on lipoprotein composition within a high cholesterol/high saturated fat diet in aged rats. Chia oil- and HxT-enriched pork partially arrested the negative effects of the atherogenic diet. Results regarding VLDL clearly suggest the reduced presence of the atherogenic cholesterol-enriched VLDL. These results are important given the large intake of meat and meat products in developed countries [1]. The benefits observed are even more relevant considering the comparisons between the experimental animals’ age and the possible effect in elderly people, suggesting the convenience to test this improved pork in elderly people affected by nonalcoholic steatohepatitis and at CVD risk. More studies are necessary in order to characterize other parts of the mechanism by which Chia oil or HxT improve the lipoprotein profile in this nonalcoholic steatohepatitis animal model. Despite the positive results observed, this study presents some possible restrictions: (1) only one dose of Chia oil and HxT were tested and (2) the study was executed only on 1-year-old male rats.

In conclusion, results support the advantage to include Chia oil or HxT in pork products to reduce the negative effect of high meat product consumption in the scenario of an atherogenic diet.

## Figures and Tables

**Table 1:** Composition of the experimental diets.

	C	HC	CHIA	HxT
Protein (%)	21.6	21.6	21.6	21.6
Fat (%)	16.9	16.6	16.6	16.6
Cholesterol (g/kg)	0.14	16.3	16.3	16.3
SFA/MUFA/PUFA	2.36/2.46/1	2.36/2.46/1	0.27/0.33/1	2.36/2.46/1
Energy content, (MJ/kg) ^1^	18.6	18.3	18.3	18.3
Ingredients (g/kg)				
Cornstarch	226.1	211.0	211.0	211.0
Casein	125.6	125.6	125.6	125.6
Maltodextrine	86.0	86.0	86.0	86.0
Sucrose	217.8	217.8	217.8	217.8
Soybean oil	44.0	44.0	44.0	44.0
Cellulose	31.4	31.4	31.4	31.4
AIN-93MX mineral mix ^2^	44.0	44.0	44.0	44.0
AIN-93VX vitamin mix ^3^	6.3	6.3	6.3	6.3
L-Cystine	1.9	1.9	1.9	1.9
Cholesterol	0	12.6	12.6	12.6
Cholic acid	0	2.5	2.5	2.5
Restructured pork ^4^	217.0	217.0	217.0	217.0

C, control-RP diet; HC, cholesterol-enriched, high-saturated/high-cholesterol, control-RP diet; HxT, hydroxytyrosol-RP, cholesterol-enriched, high-saturated/high-cholesterol diet; CHIA, Chia oil-RP, cholesterol-enriched, high-saturated/high-cholesterol diet. ^1^ Data calculated considering as energy equivalent for carbohydrates: 16.73 kJ/g (4.0 kcal/g); fat, 37.65 kJ/g (9.0 kcal/g); protein, 16.73 kJ/g (4.0 kcal/g). ^2,3^ As reported by Reeves et al. [23]. ^4^ Supplementary information.

**Table 2:** Feed intake, body weight, adipose tissue weight, fecal and cholesterol excretion, and dietary digestibility.

	C	HC	CHIA	HxT	ANOVA
Initial body weight (g)	539 ± 21.6	510 ± 39.1	493 ± 51.68	431 ± 34.2	0.094
Final body weight (g)	657 ± 55.1 ^a^	541 ± 29.4 ^b^	522 ± 16.3 ^b^	455 ± 44.2 ^c^	<0.001
Percent of body weight gain (% g/g)	21.9 ± 3.57 ^a^	6.08 ± 0.85 ^b^	5.89 ± 0.32 ^b^	5.81 ± 0.89 ^b^	<0.001
Adipose tissue weight (g) *	47.3 ± 9.96 ^a^	32.8 ± 4.53 ^b^	26.7 ± 5.38 ^b^	23.4 ± 5.52 ^b^	<0.001
Adiposomatic index ^1^	7.20 ± 1.08	6.06 ± 1.28	5.11 ± 0.98	5.14 ± 0.88	0.096
Feed intake (g/week)	119 ± 2.45	116 ± 7.85	120 ± 9.97	110 ± 7.79	0.058
Cholesterol intake (g/week)	0.08 ± 0.01 ^b^	1.6 ± 0.13 ^a^	1.6 ± 0.13 ^a^	1.6 ± 0.13 ^a^	<0.001
Fecal excretion (g/week)	5.22 ± 1.42 ^b^	8.90 ± 0.72 ^a^	8.07 ± 1.49 ^a^	8.43 ± 0.92 ^a^	<0.001
Fecal fat (mg/g dry matter)	181 ± 66.9 ^b^	249 ± 42.5 ^b^	671 ± 37.6 ^a^	199 ± 31.0 ^b^	<0.001
Fecal cholesterol (mg/g dry matter)	1.81 ± 0.88 ^b^	44.5 ± 4.88 ^a^	40.3 ± 12.12 ^a^	44.3 ± 3.51 ^a^	<0.001
Dietary digestibility ^2^	0.94 ± 0.01 ^a^	0.91 ± 0.01 ^b^	0.92 ± 0.01 ^a,b^	0.89 ± 0.01 ^b^	<0.001

C, control-RP diet; HC, cholesterol-enriched, high-saturated/high-cholesterol, control-RP diet; HxT, hydroxytyrosol-RP cholesterol-enriched, high-saturated/high-cholesterol diet; CHIA, Chia oil-RP, cholesterol-enriched, high-saturated/high-cholesterol diet. Mean values ± SD bearing different letters within a row indicate significant differences between groups (*p* < 0.05; a > b > c) (one way ANOVA followed by the T2 Tamhane post-hoc test). * Adipose tissue (mesenteric, epididymal, and perirenal). ^1^ Adiposomatic index = (adipose tissue weight/final body weight) × 100. ^2^ Dietary digestibility = (feed intake − fecal excretion)/feed intake.

**Table 3:** Plasma and liver lipid composition and atherogenic index.

	C	HC	CHIA	HxT	ANOVA
Plasma total cholesterol (mmol/L)	2.52 ± 0.29 ^b^	3.30 ± 0.29 ^a,c^	2.66 ± 0.36 ^b,c^	3.01 ± 0.24 ^a^	<0.001
Plasma triglycerides (mmol/L)	0.72 ± 0.11 ^b^	0.98 ± 0.06 ^a^	0.48 ± 0.06 ^c^	0.47 ± 0.04 ^c^	<0.001
Plasma phospholipids (mmol/L)	1.58 ± 0.07 ^b^	1.69 ± 0.19 ^a^	1.14 ± 0.18 ^c^	1.46 ± 0.06 ^b^	<0.001
Plasma total lipids (mg/dL) ^1^	282 ± 28.8 ^b^	336 ± 22.8 ^a^	235 ± 27.9 ^b^	287 ± 9.3 ^b^	<0.001
Plasma free fatty acids (mmol/L)	0.28 ± 0.06 ^b^	0.35 ± 0.05 ^a^	0.15 ± 0.03 ^c^	0.16 ± 0.05 ^c^	<0.001
Atherogenic index ^2^	0.14 ± 0.13 ^b^	1.36 ± 0.31 ^a^	1.42 ± 0.43 ^a^	1.22 ± 0.41 ^a^	<0.001
Total liver cholesterol (µmol)	277 ± 69.2 ^c^	771 ± 108 ^a^	665 ± 135 ^a^	456 ± 108 ^b^	<0.001

C, control-RP diet; HC, cholesterol-enriched, high-saturated/high-cholesterol, control-RP diet; HxT, hydroxytyrosol-RP, cholesterol-enriched, high-saturated/high-cholesterol diet; CHIA, Chia oil-RP, cholesterol-enriched, high-saturated/high-cholesterol diet. Mean values ± SD bearing different letters within a row indicate significant differences between groups (*p* < 0.05; a > b > c) (one way ANOVA followed by the T2 Tamhane post-hoc test). ^1^ Total lipids = total cholesterol + triglycerides + phospholipids. ^2^ Atherogenic index = (plasma total cholesterol − HDL cholesterol)/HDL cholesterol.

**Table 4:** Plasma lipoprotein composition.

	C	HC	CHIA	HxT	ANOVA
VLDL					
Cholesterol (mmol/L)	0.09 ± 0.01 ^b^	0.55 ± 0.21 ^a^	0.22 ± 0.13 ^a,b^	0.14 ± 0.09 ^b^	<0.001
Triglycerides (mmol/L)	0.35 ± 0.09 ^b^	0.56 ± 0.11 ^a^	0.25 ± 0.09 ^b^	0.18 ± 0.04 ^b^	<0.001
Phospholipids (mmol/L)	0.13 ± 0.03 ^b^	0.46 ± 0.08 ^a^	0.16 ± 0.08 ^b^	0.19 ± 0.11 ^b^	<0.001
Total lipids (mg/dL) ^1^	43.8 ± 9.23 ^b^	108 ± 25.57 ^a^	42.6 ± 1.52 ^b^	35.6 ± 9.61 ^b^	<0.001
Protein (mg/dL)	5.17 ± 0.21 ^c^	15. 5 ± 1.44 ^a^	8.63 ± 1.53 ^b^	10.0 ± 3.29 ^b^	<0.001
IDL + LDL					
Cholesterol (mmol/L)	0.20 ± 0.04 ^b^	1.30 ± 0.49 ^a^	1.23 ± 0.27 ^a^	1.60 ± 0.18 ^a^	<0.001
Triglycerides (mmol/L)	0.10 ± 0.05 ^a,b^	0.18 ± 0.04 ^a^	0.09 ± 0.03 ^b^	0.06 ± 0.01 ^b^	<0.001
Phospholipids (mmol/L)	0.11 ± 0.03 ^b^	0.45 ± 0.11 ^a^	0.38 ± 0.07 ^a^	0.58 ± 0.11 ^a^	<0.001
Total lipids (mg/dL) ^1^	25.5 ± 5.99 ^b^	99.2 ± 26.3 ^a^	85.0 ± 6.52 ^a^	110 ± 3.63 ^a^	<0.001
Protein (mg/dL)	6.11 ± 1.32 ^b^	28.9 ± 5.53 ^a^	27.5 ± 5.21 ^a^	33.8 ± 2.88 ^a^	<0.001
HDL					
Cholesterol (mmol/L)	2.2 ± 0.25 ^a^	1.4 ± 0.31 ^b^	1.21 ± 0.39 ^b^	1.62 ± 0.29 ^b^	<0.001
Triglycerides (mmol/L)	0.21 ± 0.10	0.12 ± 0.04	0.11 ± 0.06	0.14 ± 0.05	0.059
Phospholipids (mmol/L)	1.1 ± 0.13 ^a^	0.65 ± 0.10 ^b^	0.52 ± 0.17 ^b^	0.68 ± 0.09 ^b^	<0.001
Total lipids (mg/dL) ^1^	158 ± 51.8 ^a^	93.3 ± 17.8 ^b^	76.7 ± 26.6 ^b^	105 ± 17.2 ^b^	<0.001
Protein (mg/dL)	121 ± 37.4 ^a^	71.7 ± 14.1 ^b^	68.7 ± 17.4 ^b^	78.9 ± 17.1 ^b^	0.016

C, control-RP diet; HC, cholesterol-enriched, high-saturated/high-cholesterol, control-RP diet; HxT, hydroxytyrosol-RP, cholesterol-enriched, high-saturated/high-cholesterol diet; CHIA, Chia oil-RP, cholesterol-enriched, high-saturated/high-cholesterol diet. VLDL, very low-density lipoproteins; IDL, intermediate-density lipoproteins; LDL, low-density lipoproteins; HDL, high-density lipoproteins. Mean values ± SD bearing different letters within a row indicate significant differences between groups (*p* < 0.05; a > b > c) (one way ANOVA followed by the T2 Tamhane post-hoc test). ^1^ Total lipids = cholesterol + triglycerides + phospholipids. To transform mmol/L into mg/dL, values should be multiplied by 38.7, 89 and 75 for cholesterol, triglycerides and phospholipids.

**Table 5:** Liver *Ldlr* and SREBP-1c expression.

	C	HC	CHIA	HxT	ANOVA
*Ldlr*	1.00 ± 0.07 ^a^	0.33 ± 0.15 ^b^	0.48 ± 0.14 ^b^	0.44 ± 0.13 ^b^	<0.001
SREBP-1c	1.00 ± 0.19 ^b^	1.33 ± 0.14 ^a^	0.55 ± 0.05 ^c^	0.65 ± 0.06 ^c^	<0.05

C, control-RP diet; HC, cholesterol-enriched, high-saturated/high-cholesterol, control-RP diet; HxT, hydroxytyrosol-RP, cholesterol-enriched, high-saturated/high-cholesterol diet; CHIA, Chia oil-RP cholesterol-enriched, high-saturated/high-cholesterol diet. Mean values ± SD bearing different letters within a row indicate significant differences between groups (*p* < 0.05; a > b > c) (one way ANOVA followed by the T2 Tamhane post-hoc test). Values expressed as relative to control (C).

**Table 6:** Pearson product-moment correlation between variables.

	Cholesterol Intake (g/week)	Fecal Fat (mg/g Dry Matter)	Fecal Cholesterol (mg/g Dry Matter)	Plasma Total Cholesterol (mmol/L)	Total Liver Cholesterol (µmol)	Plasma Triglycerides (mmol/L)	Plasma Phospholipids (mmol/L)	Plasma Total Lipids (mg/dL)	Plasma Free Fatty Acids (mmol/L)	Atherogenic Index	Ldlr Gene Expression	SREBP-1c Protein Expression	VLDL Total Mass (mg/dL)	IDL + LDL Total Mass (mg/dL)	HDL Total Mass (mg/dL)
Percent of body weight gain (% g/g)	−0.503 **	−0.632 ***			−0.524 **										0.607 ***
Adipose tissue weight (g)	−0.730 ***		−0.766 ***			0.525 **	0.539 **		0.620 ***	−0.593 ***	0.744 ***	0.521 **		−0.675 ***	0.700 ***
Cholesterol intake (g/week)			−0.952 ***	0.533 **	0.676 ***					−0.707 ***	−0.857 ***			−0.870 ***	−0.724 ***
Fecal fat (mg/g dry matter)							−0.800 ***	−0.732 ***							
Fecal cholesterol (mg/g dry matter)					0.697 ***						−0.872 ***			−0.807 ***	−0.711 ***
Plasma total cholesterol (mmol/L)								0.561 **						−0.807 ***	
Total liver cholesterol (µmol)											-0.629 ***				−0.532 **
Plasma triglycerides (mmol/L)							0.657 ***	0.745 ***	0.766 ***			0.761 ***	0.814 ***		
Plasma phospholipids (mmol/L)								0.878 ***	0.641 **			0.712 ***			
Plasma total lipids (mg/dL)									0.648 **			0.679 ***	0.598 **		
Plasma free fatty acids (mmol/L)												0.762 ***			
Atherogenic index														0.627 **	−0.713 ***
SREBP-1c protein expression													0.697 ***		

Values correspond to regression adjustments. ** *p* < 0.01; *** *p* < 0.001.

## Supplementary Materials

The following are available online at http://www.mdpi.com/2072-6643/10/12/1830/s1. Detailed data on restructured pork composition are available on Table S1.
