# Supplementary material for: Lipoprotein Profile in Aged Rats Fed Chia Oil- or Hydroxytyrosol-Enriched Pork in High Cholesterol/High Saturated Fat Diets
Source: Nutrients. 2018 Nov 26;10(12):1830. doi: 10.3390/nu10121830 (PMC6316572; doi:10.3390/nu10121830)
Supplement: Supplementary file 1 [file nutrients-10-01830-s001.pdf]

Table S1. Composition of the Restructured Pork (RP)

	**C and HC**	**CHIA**	**HxT**
Protein (%)	15.6	15.6	15.6
Fat (%)	21.9	21.9	21.9
Water (%)	62.5	62.5	62.5
Cholesterol (*g/kg*)	0.65	0.65	0.65
SFA/MUFA/PUFA ratio	3.99/4.32/1	0.3/0.37/1	3.99/4.32/1
Ingredients (g/kg)			
Lean pork	801.3	801.3	799.5
Lard	152.2	0.0	152.2
NaCl	5.1	5.1	5.1
Sodium nitrite	1.2	1.2	1.2
STP	0.1	0.1	0.1
Hydroxytyrosol	0.0	0.0	3.6
Chia oil	0.0	152.2	0.0
Water	40.1	40.1	38.3

C, control RP diet; HC, Cholesterol enriched high-saturated/high-cholesterol control RP diet; HxT, hydroxytyrosol RP Cholesterol enriched high-saturated/high-cholesterol diet; CHIA, chia oil RP Cholesterol enriched high-saturated/high-cholesterol diet.
